# Glutathione-Mediated Redox Regulation of Immune Dysfunction in COVID-19 and Tuberculosis

**DOI:** 10.3390/antiox15020214

**Published:** 2026-02-06

**Authors:** John Dawi, Scarlet Affa, Yura Misakyan, Edgar Gonzalez, Stephen Affa, Vishwanath Venketaraman

**Affiliations:** 1 College of Osteopathic Medicine of the Pacific, Western University of Health Sciences, Pomona, CA 91766, USA; john.dawi@westernu.edu (J.D.); yura.misakyan@westernu.edu (Y.M.); edgar.gonzalez@westernu.edu (E.G.); 2 Department of Biochemistry and Chemistry, The College, University of Los Angeles California, 405 Hilgard Avenue, Los Angeles, CA 90095, USA; scarleta@ucla.edu (S.A.); stephena1@ucla.edu (S.A.)

**Keywords:** glutathione, redox homeostasis, immune dysregulation, tuberculosis, COVID-19

## Abstract

Tuberculosis and coronavirus disease 2019, also known as COVID-19, remain major global health challenges that disproportionately affect individuals with metabolic disorders, chronic inflammation, and limited access to healthcare. Although these diseases are caused by different pathogens, they share important host-related determinants of severity, including immune dysfunction, oxidative stress, endothelial injury, and maladaptive inflammatory responses. Glutathione, the primary intracellular antioxidant and a key regulator of redox balance, has emerged as an important host factor connecting these processes across infectious diseases. This review integrates experimental, translational, and clinical evidence supporting the role of glutathione in regulating immune function, oxidative stress, and tissue damage in tuberculosis and COVID-19. In tuberculosis, glutathione deficiency compromises macrophage antimicrobial activity, disrupts granuloma structure, and alters T helper cell responses, leading to impaired immune containment and disease progression. In COVID-19, reduced glutathione levels are associated with redox imbalance, excessive cytokine signaling, endothelial dysfunction, and thromboinflammatory complications, especially in high-risk populations. In both diseases, glutathione depletion reduces host resilience and increases vulnerability to severe outcomes through shared immune and vascular pathways. By unifying disease-specific findings within a host-directed framework, this review highlights glutathione and redox signaling as common vulnerability pathways that help explain overlapping risk profiles for severe tuberculosis and COVID-19. It also places glutathione biology within the broader context of host-directed immunotherapy, emphasizing its potential role in prevention-focused and resilience-based strategies that complement pathogen-targeted treatments. Although current evidence does not support simple claims of disease prevention, it provides strong mechanistic justification for further investigation of glutathione as a modifiable host factor in high-risk populations.

## 1. Introduction

Tuberculosis (TB) remains one of the leading infectious causes of morbidity and mortality worldwide despite decades of global control efforts. Recent global analyses demonstrate that TB continues to impose a substantial disease burden, with millions of new cases annually and persistent mortality, particularly in low- and middle-income countries and among vulnerable populations [1]. Although advances in diagnostics and antimicrobial therapy have improved outcomes, progress toward TB elimination has been uneven, and the persistence of latent infection and drug-resistant strains continues to undermine global control strategies [2]. These challenges highlight the need for complementary approaches that address host susceptibility and immune dysfunction in addition to pathogen-directed therapies.

Certain populations are disproportionately affected by TB due to underlying metabolic, immunologic, and socioeconomic vulnerabilities. Diabetes mellitus, for example, has emerged as a major global risk factor for active TB, with systematic reviews demonstrating a significantly higher prevalence of diabetes among patients with TB compared to the general population [3,4]. Immunocompromised states, chronic disease, malnutrition, and advanced age further increase the risk of disseminated and severe TB, underscoring the role of impaired host defenses in disease progression [5]. Collectively, these data support the classification of TB as not only an infectious disease but also a condition strongly shaped by host immune and metabolic status.

The emergence of coronavirus disease 2019 (COVID-19) has further exposed and amplified vulnerabilities within these same high-risk populations. Large population-based analyses and meta-analyses have consistently shown that older age, diabetes, obesity, cardiovascular disease, and other chronic conditions are strongly associated with severe COVID-19 and increased mortality [6,7,8,9]. These overlapping risk factors suggest shared pathophysiologic pathways that predispose individuals to both severe viral and bacterial infections, including dysregulated immune responses and heightened oxidative stress.

Importantly, TB and COVID-19 do not exist in isolation. Growing evidence indicates that coinfection with Mycobacterium tuberculosis and severe acute respiratory syndrome coronavirus 2 (SARS-CoV-2) is associated with worse clinical outcomes than either disease alone. Systematic reviews and meta-analyses have documented the global prevalence of TB–COVID-19 coinfection and have reported increased mortality and complications among affected patients, particularly in regions with high TB endemicity [10,11]. These findings reinforce the urgency of developing interventions that can enhance host resilience in populations at risk for both diseases.

In this context, host-directed therapies have gained increasing attention as adjunctive strategies to improve outcomes in infectious diseases. Rather than targeting the pathogen directly, host-directed approaches aim to modulate immune responses, reduce pathological inflammation, and enhance antimicrobial defenses. In TB, host-directed therapies have been proposed as a means to improve treatment efficacy, limit tissue damage, and address the heterogeneity of host immune responses that influence disease severity and outcomes [12]. Such strategies may be particularly valuable in high-risk populations with underlying immune dysfunction.

Glutathione, a central intracellular antioxidant and regulator of redox homeostasis, has emerged as a biologically plausible candidate for host-directed intervention in infectious diseases. Glutathione plays a critical role in maintaining immune cell function, regulating inflammatory signaling, and protecting against oxidative stress. The emerging literature has linked glutathione deficiency to increased susceptibility to severe COVID-19, with reviews suggesting that impaired glutathione availability may contribute to excessive inflammation, endothelial injury, and immune dysregulation observed in severe disease [13,14,15]. While these findings do not establish causality, they provide a mechanistic framework through which glutathione status may influence disease severity.

Parallel evidence has implicated glutathione in host defense against Mycobacterium tuberculosis. Recent reviews have highlighted the role of glutathione in macrophage function, cytokine balance, and antimicrobial activity, particularly in populations with compromised immunity [16]. Reduced glutathione levels have been observed in individuals with chronic disease and immune dysfunction, suggesting that deficiency may impair the host’s ability to control intracellular pathogens such as *M. tuberculosis*. Together, these data support further investigation into glutathione as a modulator of immune responses relevant to both TB and COVID-19.

Given the shared risk factors, overlapping pathophysiology, and disproportionate burden of TB and COVID-19 among high-risk populations, exploring the role of glutathione as a preventive or adjunctive strategy is both timely and biologically grounded. Understanding how glutathione influences immune responses across these diseases may inform future host-directed interventions aimed at reducing disease severity and improving outcomes in vulnerable populations.

## 2. Materials and Methods

### 2.1. Literature Search Strategy

A structured literature search was conducted using the PubMed database to identify peer-reviewed studies examining the role of glutathione in immune regulation, redox homeostasis, and inflammation-related disease processes. The search included articles published in English up to the time of manuscript preparation and was not restricted by geographic location.

Search terms were selected to capture both mechanistic and translational aspects of glutathione biology and included combinations of the following keywords: glutathione, redox homeostasis, oxidative stress, immune function, inflammation, ferroptosis, GPX4, SLC7A11, cytokine signaling, innate immunity, and adaptive immunity. Boolean operators (“AND,” “OR”) were applied to refine the search strategy and ensure comprehensive retrieval of relevant studies.

### 2.2. Study Selection and Eligibility Criteria

Study selection was performed in accordance with PRISMA guidelines using a two-stage screening process. Titles and abstracts were initially screened for relevance, followed by full-text review of potentially eligible articles.

Inclusion Criteria were: 

Original research articles, narrative reviews, or systematic reviews.

Studies investigating glutathione metabolism, antioxidant defense systems, or redox-dependent signaling pathways.

Studies examining immune cell regulation, inflammatory responses, or disease mechanisms influenced by oxidative stress.

Experimental (in vitro or in vivo), translational, and clinically relevant human studies.

Exclusion Criteria were:

Publications not primarily focused on glutathione or redox biology.

Studies lacking relevance to immune regulation or inflammatory mechanisms.

Editorials, commentaries, conference abstracts, and non-peer-reviewed sources.

Non-English-language publications.

### 2.3. Data Extraction and Qualitative Synthesis

Eligible studies underwent full-text review, and relevant data were extracted focusing on molecular mechanisms, immune signaling pathways, and disease associations related to glutathione and redox balance. Emphasis was placed on studies describing glutathione-dependent regulation of reactive oxygen species, NF-κB signaling, inflammasome activation, ferroptosis, and immune cell function.

Due to heterogeneity in study design, methodology, and reported outcomes, a qualitative narrative synthesis approach was employed rather than a quantitative meta-analysis. Findings were integrated to identify shared biological themes and redox-immune pathways across multiple disease contexts and to support the conceptual framework illustrated in the schematic figures.

### 2.4. PRISMA Flow Description

The initial PubMed search yielded approximately 140 records. After removal of duplicates and screening of titles and abstracts, 78 articles were selected for full-text review. Of these, 28 studies were excluded due to insufficient relevance to glutathione-mediated immune regulation or lack of mechanistic detail. A total of 50 peer-reviewed studies met inclusion criteria and were incorporated into the final qualitative synthesis.

### 2.5. Final Study Inclusion

The final selection of 50 studies formed the basis for the mechanistic framework and schematic figures presented, highlighting glutathione as a central regulator of immune function and redox homeostasis across diverse disease states.

## 3. Glutathione as a Central Regulator of Immune Function and Redox Homeostasis

**Figure 1:**
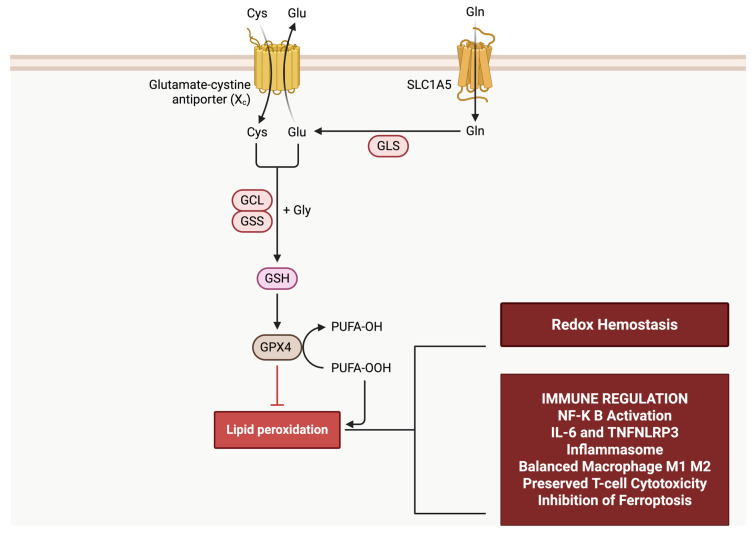
This schematic illustrates the central role of intracellular glutathione (GSH) in maintaining redox homeostasis and regulating immune function. Cysteine uptake through system Xc^−^ (SLC7A11) supports intracellular GSH synthesis, which fuels GPX4 and other glutathione peroxidases to detoxify reactive oxygen species. Adequate redox control suppresses NF-κB activation, limits pro-inflammatory cytokine production, inhibits NLRP3 inflammasome activation, preserves balanced macrophage polarization, maintains T cell effector function, and prevents ferroptotic cell death.

Glutathione (GSH) is a low-molecular-weight thiol that functions as a dominant intracellular redox buffer and signaling modifier in mammalian cells, as shown in Figure 1. In immune cells, GSH is not simply a passive antioxidant pool but an active determinant of metabolic fitness, signal transduction fidelity, and effector programming, because immune activation requires controlled redox flux rather than indiscriminate suppression of reactive species. This is particularly relevant in high-risk hosts, where baseline oxidative stress and altered metabolism can constrain immune cell function and bias inflammatory outputs, creating a permissive landscape for severe infection.

### 3.1. De Novo Glutathione Synthesis Is an Immune Cell Requirement, Not an Optional Accessory

A key concept for this project is that immune competence depends on the ability of immune cells to synthesize GSH de novo under demand. In T lymphocytes, genetic and metabolic work demonstrates that the capacity to generate GSH is tightly linked to the ability to mount inflammatory responses. Specifically, de novo GSH synthesis primes T cell metabolism to support inflammatory function, connecting intracellular thiol availability with the metabolic transitions that accompany activation and effector differentiation [17]. This work positions GSH as a gating factor for T cell inflammatory programming rather than a downstream marker of “oxidative stress.”

Mechanistic studies further show that the metabolic routes feeding GSH synthesis shape T cell fate decisions. Glutamine-derived pathways that supply precursors for glutathione synthesis influence lineage choice, indicating that the immunologic outputs of T cells depend in part on whether metabolic substrates can be routed toward GSH generation and redox control [18]. In other words, the same metabolic networks that fuel proliferation also establish redox conditions that bias differentiation, creating a mechanistic bridge between nutrient availability, intracellular thiol chemistry, and adaptive immune polarization [18].

Transcriptional control of these programs is also relevant. ATF4-dependent regulation of CD4+ T cell responses ties cellular stress adaptation and metabolic reprogramming to effective immune function, including the maintenance of antioxidant capacity and redox balance required for sustained activation [19]. This provides a conceptual framework for why physiologic and disease-associated stress states can impair T cell responses: when stress response programs cannot maintain appropriate redox homeostasis, signaling and effector function degrade [19].

Beyond metabolic and transcriptional regulation, T cell activation is intrinsically redox-sensitive at the signaling level. Reactive oxygen species participate in T cell signal transduction, and the intracellular antioxidant network, including glutathione, shapes the amplitude and duration of these signals [20]. The implication for a preventive framework is not that immune responses require maximal ROS suppression; rather, appropriate glutathione availability supports “signal-competent” redox tone that permits activation while limiting destructive oxidative injury [20].

Human T cell functional data reinforce the point that perturbing glutathione alters immune behavior. In primary human T cells, experimentally depleting glutathione suppresses inflammatory responses, demonstrating that intact glutathione pools are required to sustain specific activation phenotypes [21]. This supports using glutathione status as a biologically meaningful variable when discussing host susceptibility in populations at risk for TB and COVID-19.

### 3.2. Glutathione Links Immune Signaling to Immunometabolism

The immune system’s effector programs are built on coordinated metabolic shifts. GSH sits at the intersection of these shifts because it both depends on and regulates cellular metabolism. In activated T cells, work demonstrating that GSH primes metabolism for inflammation implies a bidirectional relationship: metabolic reprogramming fuels GSH synthesis, and adequate GSH supports metabolic pathways needed for effector function [17]. This reciprocal dependence is critical in high-risk populations characterized by metabolic disease, because constraints on substrate availability and mitochondrial function can impair both GSH synthesis and immune cell effector performance [17,18].

Studies connecting glutamine metabolism and glutathione synthesis to T cell lineage choice illustrate how metabolic routing affects differentiation outcomes, which has downstream consequences for host defense against intracellular pathogens where specific T helper programs are protective [18]. In practical terms, if a high-risk host has impaired nutrient handling or altered amino acid metabolism, this may translate to altered glutathione availability and thus altered immune polarization, potentially weakening protective T cell responses [18].

At the signaling layer, redox-mediated tuning of T cell activation is a mechanistic explanation for why glutathione depletion can blunt inflammatory responses in primary human T cells [20,21]. Redox-sensitive nodes in receptor-proximal signaling, transcription factor activation, and effector gene transcription require a controlled thiol environment, which glutathione helps maintain [20,21]. This provides a coherent mechanistic rationale to place glutathione biology early in the argument as a plausible host-directed lever.

### 3.3. Innate Immunity: Macrophage Inflammatory Programming and Glutathione-Dependent Control

Innate immune cells, particularly macrophages, are central to early defense against infection and are also major drivers of inflammatory tissue injury. Multiple lines of evidence support that glutathione levels and glutathione-linked metabolism influence macrophage activation states and cytokine outputs.

Experimental work demonstrates that glutathione can modulate macrophage polarization and immune-stimulatory activity, indicating that macrophage phenotype is sensitive to the glutathione redox environment [22]. This is directly relevant to TB pathogenesis where macrophage microbicidal activity and inflammatory tone determine containment versus progression, and to COVID-19 where macrophage-driven inflammation contributes to severe disease phenotypes [22].

Immunometabolic coupling is also evident in macrophages through amino acid metabolism. Serine metabolism has been shown to support macrophage IL-1β production via glutathione synthesis, linking a specific nutrient pathway to a defined inflammatory cytokine output through a glutathione-dependent mechanism [23]. This is important for Section 2 because it supplies a concrete, mechanistic example of how glutathione is embedded in innate immune cytokine biology, not merely an antioxidant “background factor” [23].

Pharmacologic and biochemical approaches further highlight glutathione’s role in inflammatory signaling pathways. A glutathione derivative (GSH-C4) has been characterized as exerting anti-inflammatory effects through inhibition of NF-κB signaling in inflammatory models [24]. While such findings do not equate to clinical prevention, they provide mechanistic support that glutathione-linked interventions can modulate canonical inflammatory transcriptional pathways that govern cytokine production and immune activation intensity [24].

### 3.4. Protein S-Glutathionylation: A Key Mechanism by Which Glutathione Regulates Immune Signaling

To cover glutathione biology comprehensively, it is essential to include *S*-glutathionylation, the reversible post-translational modification in which glutathione forms a mixed disulfide with protein cysteine residues. This mechanism provides a direct biochemical route by which glutathione availability can alter protein function, signaling pathway dynamics, and cellular activation thresholds. Contemporary mechanistic reviews describe *S*-glutathionylation as a major mode of redox regulation, affecting a wide array of proteins and serving as a functional switch that couples redox state to cellular behavior [25].

Importantly, *S*-glutathionylation is not restricted to “stress response” proteins but extends into metabolic control, with evidence describing these reactions as broad inhibitors or modulators of metabolism [26]. For immune cells, this is highly relevant because metabolic pathway flux directly determines activation, cytokine production, and effector functions; thus glutathione-mediated control of protein thiols can translate into functional immune phenotypes [26].

Direct immune cell evidence exists for *S*-glutathionylation, influencing lineage and differentiation programs. GSTP-mediated *S*-glutathionylation has been shown to affect dendritic cell differentiation and metabolism, linking glutathione-dependent thiol modification to antigen-presenting cell development and function [27]. Because dendritic cells shape T cell priming and polarization, a glutathione-dependent mechanism at the level of dendritic differentiation provides a plausible upstream pathway through which host redox status can influence adaptive immunity [27].

Together, these findings justify viewing glutathione as a signaling regulator: by enabling reversible thiol modifications, glutathione integrates the cellular redox state with immune activation, differentiation, and inflammatory amplitude [25,26,27]. In high-risk populations where oxidative stress is chronically elevated, shifts in glutathione pools and thiol–disulfide equilibrium may plausibly alter the extent and pattern of *S*-glutathionylation, thereby changing immune responsiveness at multiple levels [25,26,27].

### 3.5. Translational Feasibility: Manipulating Glutathione in Humans

A preventive framework also requires biological feasibility: can glutathione status be modified in humans in a measurable way? Randomized controlled data indicate that oral glutathione can increase glutathione-related measures in humans, providing proof of principle that systemic glutathione biology is modifiable through supplementation strategies under controlled conditions [28]. While such trials do not establish disease prevention, they strengthen the translational foundation for considering glutathione as a modifiable host factor, particularly when paired with mechanistic immune evidence showing glutathione-dependent control of T cell activation, macrophage cytokines, and redox signaling [17,18,19,20,21,22,23,24,28].

### 3.6. Section Synthesis: Why Glutathione Belongs in a Host-Directed Framework for High-Risk Infection

The collective message of this section is mechanistic rather than promotional. Across adaptive immunity, glutathione de novo synthesis and glutathione-dependent redox control are required for robust T cell inflammatory programming, differentiation decisions, and maintenance of activation-competent signaling [17,18,19,20,21]. Across innate immunity, glutathione influences macrophage polarization, cytokine production pathways such as IL-1β, and canonical inflammatory signaling networks including NF-κB [22,23,24]. At the biochemical core, glutathione exerts regulatory control through reversible *S*-glutathionylation, shaping both signaling and metabolism, with specific evidence in antigen-presenting cell differentiation [25,26,27]. Finally, human supplementation trials support the feasibility of modulating glutathione biology, which justifies continued investigation in the context of infectious disease susceptibility and severity in high-risk populations [28].

This mechanistic foundation sets up the next disease-focused sections by defining how glutathione status can plausibly influence immune competence and inflammatory balance, the two host dimensions most consistently implicated in both TB progression and severe COVID-19.

## 4. Glutathione and Tuberculosis Pathophysiology in High-Risk Populations

Tuberculosis is a chronic intracellular infection in which disease progression reflects a failure of host immune containment rather than unchecked microbial replication alone. Successful control of *Mycobacterium tuberculosis* requires coordinated innate and adaptive immune responses that are sustained over long periods and executed under conditions of persistent inflammatory and oxidative stress. Increasing experimental and translational evidence indicates that intracellular glutathione availability is a central determinant of these responses, influencing macrophage antimicrobial activity, granuloma integrity, cytokine balance, and the quality of Th1-mediated immunity. In high-risk populations, including individuals with metabolic disease, chronic inflammation, or immune dysregulation, glutathione deficiency emerges as a biologically plausible contributor to tuberculosis susceptibility and disease severity.

### 4.1. Glutathione Deficiency, Oxidative Stress, and Impaired Host Containment of M. tuberculosis

Macrophages serve as both the primary host cells for *M. tuberculosis* and the principal effectors of early antimicrobial defense, as shown in Table 1. Effective macrophage responses require a tightly regulated redox environment, as excessive oxidative stress can impair phagolysosomal function, disrupt signaling pathways, and promote tissue damage rather than bacterial killing. Experimental models of glutathione depletion demonstrate that reduced intracellular GSH levels lead to heightened oxidative stress within immune cells, accompanied by impaired control of intracellular mycobacteria and increased bacterial survival [29].

In murine systems, glutathione deficiency is associated with defective granuloma formation, characterized by impaired cellular organization and reduced ability to contain *M. tuberculosis* within structured immune aggregates [29]. Because granulomas represent a physical and immunologic barrier that limits bacterial dissemination, disruption of glutathione-dependent redox balance may undermine one of the host’s most critical defense mechanisms. These findings suggest that glutathione deficiency does not merely reflect downstream oxidative injury during tuberculosis but actively contributes to immune failure at the tissue level.

The relevance of these observations is amplified in high-risk populations, where baseline oxidative stress is often elevated due to metabolic dysfunction, chronic inflammation, or coexisting disease. In such contexts, inadequate glutathione reserves may predispose macrophages to dysfunctional inflammatory responses that favor persistence of *M. tuberculosis* rather than effective containment.

### 4.2. Glutathione and Th1-Polarized Immunity in Tuberculosis

Protective immunity against tuberculosis depends heavily on sustained Th1-polarized adaptive responses, particularly the production of interferon-γ and interleukin-12, which activate macrophage antimicrobial pathways and support long-term containment of infection. Multiple studies demonstrate that glutathione availability directly influences the generation and maintenance of this cytokine milieu.

In human immune cell models, liposomal glutathione supplementation restores Th1 cytokine production in cells exposed to *M. tuberculosis*, indicating that glutathione deficiency contributes to impaired adaptive immune signaling [30]. These findings are consistent with broader evidence that glutathione regulates T cell activation and cytokine gene expression through redox-sensitive signaling pathways [22]. When glutathione levels are insufficient, redox imbalance interferes with transcriptional programs required for effective Th1 differentiation and function, thereby weakening a core pillar of tuberculosis immunity.

Importantly, tuberculosis is characterized by prolonged antigen exposure and chronic immune activation, conditions under which glutathione depletion may become progressively more pronounced. Under these circumstances, even modest impairments in glutathione synthesis or recycling could translate into cumulative defects in Th1 responses, contributing to disease progression or failure of immune containment over time.

### 4.3. Glutathione and Granulomatous Immune Architecture

Granuloma formation is a defining feature of tuberculosis pathophysiology and represents a dynamic immunologic structure rather than a static barrier. Effective granulomas require coordinated interactions between macrophages, T lymphocytes, and other immune cells, as well as controlled cytokine gradients and metabolic support. Experimental studies examining glutathione supplementation demonstrate that restoring intracellular GSH levels favorably alters granulomatous responses to *M. tuberculosis* infection [31].

In both in vitro and in vivo models, glutathione supplementation enhances immune cell organization within granuloma-like structures and reduces intracellular mycobacterial burden [31]. These effects are accompanied by shifts in cytokine profiles toward a more protective pattern, suggesting that glutathione influences not only structural containment but also the functional quality of immune responses within granulomas. This is particularly relevant given that poorly organized or necrotic granulomas are associated with increased bacterial replication and disease dissemination.

The link between glutathione status and granuloma integrity provides a mechanistic explanation for why redox imbalance may predispose certain individuals to progressive or disseminated tuberculosis despite intact immune cell numbers. It also underscores the importance of metabolic and redox support in sustaining long-term immune architecture during chronic infection.

### 4.4. Glutathione as an Immunomodulatory Adjunct in Tuberculosis Models

Beyond its endogenous role in immune regulation, glutathione has been investigated as a host-directed immunomodulatory adjunct in tuberculosis models. Experimental studies characterizing glutathione as an immunoadjuvant demonstrate that supplementation enhances protective immune responses and improves control of *M. tuberculosis* when combined with antimycobacterial strategies [32]. These effects include modulation of cytokine balance, enhancement of macrophage antimicrobial activity, and reduction in intracellular bacterial survival.

The relevance of these findings is especially pronounced in metabolically compromised hosts. In models of type 2 diabetes mellitus, a well-recognized risk factor for tuberculosis, oral liposomal glutathione supplementation improves immune responses against *M. tuberculosis* and *Mycobacterium bovis* BCG, reducing intracellular mycobacterial burden and correcting cytokine imbalances associated with metabolic disease [33]. These data suggest that glutathione deficiency may represent a reversible contributor to immune dysfunction in high-risk populations rather than an immutable consequence of underlying disease.

### 4.5. Translational Implications and Human Relevance

Human studies provide additional support for the translational relevance of glutathione modulation. Oral liposomal glutathione supplementation has been shown to alter systemic oxidative stress markers and immune function in humans, demonstrating that glutathione status can be safely and measurably modified [28]. While such studies do not establish clinical efficacy against tuberculosis, they provide proof of biological feasibility that supports further investigation in at-risk populations.

Integrative reviews of glutathione biology in tuberculosis emphasize that glutathione depletion interacts with other immunoregulatory pathways, including TGF-β signaling and vitamin D-dependent immune responses, both of which are implicated in tuberculosis pathogenesis [34]. This convergence suggests that glutathione sits at a nexus of metabolic, redox, and immunologic regulation, amplifying its relevance as a host-directed factor in tuberculosis susceptibility and disease progression.

### 4.6. Section Synthesis

Taken together, the evidence supports a coherent model in which glutathione availability is a critical determinant of immune competence in tuberculosis. Glutathione deficiency promotes oxidative stress, disrupts macrophage antimicrobial function, compromises granuloma integrity, and blunts Th1-polarized adaptive responses, all of which are essential for controlling *M. tuberculosis* infection [29,30,31,32,33,34]. Conversely, restoring glutathione levels enhances cytokine signaling, improves immune architecture, and reduces mycobacterial burden in experimental models, with emerging relevance for high-risk human populations [28,30,31,32,33].

This mechanistic foundation provides a strong rationale for examining glutathione as a host-directed factor in tuberculosis prevention and disease modulation. It also establishes a conceptual bridge to the next section, which will examine how similar glutathione-dependent immune vulnerabilities may influence susceptibility and outcomes in COVID-19, particularly in populations already burdened by metabolic and inflammatory stress.

## 5. Glutathione and COVID-19 Pathophysiology in High-Risk Populations

Severe acute respiratory syndrome coronavirus 2 (SARS-CoV-2) infection is characterized by a complex host–pathogen interaction in which clinical severity is determined largely by host response rather than viral replication alone. While viral entry and early replication initiate infection, progression to severe coronavirus disease 2019 (COVID-19) is driven by dysregulated immune activation, oxidative stress amplification, endothelial dysfunction, and maladaptive inflammatory signaling. Glutathione, as the principal intracellular antioxidant and redox regulator, occupies a central position at the intersection of these processes. Increasing evidence indicates that glutathione depletion is not merely a consequence of severe COVID-19 but a pre-existing and infection-exacerbated vulnerability that shapes disease trajectory, particularly in high-risk populations.

### 5.1. Baseline Glutathione Deficiency and Predisposition to Severe COVID-19

Endogenous glutathione deficiency has been proposed as a unifying factor underlying susceptibility to severe COVID-19. Glutathione is essential for maintaining intracellular redox equilibrium, preserving mitochondrial integrity, and regulating immune signaling pathways that depend on thiol–disulfide balance. Analyses of COVID-19 pathogenesis emphasize that individuals with reduced baseline glutathione reserves exhibit heightened vulnerability to SARS-CoV-2-induced oxidative stress, immune dysregulation, and inflammatory escalation [35].

This vulnerability is particularly relevant because many of the strongest epidemiologic risk factors for severe COVID-19 are independently associated with glutathione depletion. Aging is accompanied by reduced glutathione synthesis and impaired recycling, while metabolic conditions such as type 2 diabetes mellitus, obesity, and cardiovascular disease are characterized by chronic oxidative stress and diminished antioxidant capacity [36]. In these populations, SARS-CoV-2 infection superimposes an acute oxidative burden onto an already compromised redox environment, creating conditions that favor immune imbalance rather than controlled antiviral defense.

The concept that glutathione deficiency precedes infection in many severe cases reframes COVID-19 not solely as an acute viral illness but as a stress test of host redox resilience. Under this model, individuals with sufficient glutathione reserves may tolerate the oxidative and inflammatory demands of infection, whereas those with depleted reserves experience rapid progression toward immune dysregulation and tissue injury.

### 5.2. SARS-CoV-2-Induced Oxidative Stress and Collapse of Glutathione Homeostasis

SARS-CoV-2 infection induces oxidative stress through multiple converging mechanisms. Viral replication disrupts mitochondrial function, immune cell activation generates reactive oxygen species, and endothelial injury further amplifies redox imbalance. Reviews of COVID-19 redox biology consistently describe a marked shift toward oxidative stress, with depletion of reduced glutathione and impairment of glutathione recycling pathways [37].

Clinical studies corroborate these mechanistic insights. Hospitalized patients with COVID-19 demonstrate altered glutathione redox status, including reduced levels of reduced glutathione and increased markers of oxidative damage. These alterations correlate with disease severity, need for intensive care, and mortality [15,38,39]. Importantly, these redox disturbances are observed early in severe disease, suggesting that glutathione depletion participates in pathogenesis rather than representing a late epiphenomenon.

Loss of glutathione buffering capacity has profound consequences for immune regulation. Many immune signaling pathways rely on controlled redox signaling, and excessive oxidative stress disrupts this balance. In the absence of sufficient glutathione, redox-sensitive transcription factors such as NF-κB and interferon regulatory factors become dysregulated, favoring excessive cytokine production while impairing antiviral coordination [15,37,38,39]. This dual effect—hyperinflammation coupled with ineffective viral control—represents a defining feature of severe COVID-19.

### 5.3. Glutathione Depletion and Cytokine Amplification

One of the hallmarks of severe COVID-19 is a cytokine-driven inflammatory state marked by elevated IL-6, TNF-α, IL-1β, and other mediators. The magnitude and persistence of this inflammatory response are strongly influenced by intracellular redox status. Glutathione serves as a critical modulator of cytokine signaling by constraining oxidative amplification of inflammatory pathways.

When glutathione levels are depleted, oxidative stress removes inhibitory checks on pro-inflammatory signaling cascades, allowing cytokine production to become self-reinforcing [40]. Reviews focusing on glutathione in COVID-19 emphasize that adequate glutathione availability supports immune proportionality, permitting effective antiviral signaling without unchecked inflammation [36,40]. Conversely, glutathione deficiency promotes a state in which inflammation is both excessive and poorly regulated.

This phenomenon is particularly relevant to COVID-19 because sustained inflammation contributes directly to lung injury, vascular permeability, and systemic complications. The inability to resolve inflammatory signaling once viral replication declines may reflect, in part, persistent glutathione depletion and ongoing redox imbalance.

### 5.4. Endothelial Dysfunction, Immunothrombosis, and Redox Injury

COVID-19 is increasingly recognized as a vascular disease as much as a respiratory one. Endothelial cells are highly sensitive to oxidative stress, and glutathione is a primary intracellular defense against redox-mediated endothelial injury. In COVID-19, endothelial dysfunction manifests as increased permeability, activation of coagulation pathways, and microvascular thrombosis.

Analyses of COVID-19-associated immunothrombosis identify glutathione depletion as a contributor to endothelial injury and dysregulated coagulation [41]. Oxidative stress promotes endothelial activation, platelet aggregation, and expression of pro-thrombotic factors, while insufficient glutathione impairs the cell’s ability to counteract these processes. In high-risk individuals with baseline endothelial dysfunction, such as those with diabetes or cardiovascular disease, glutathione depletion may amplify vascular injury and increase the risk of thromboembolic complications.

This vascular dimension further broadens the relevance of glutathione beyond immune cells, positioning it as a systemic determinant of COVID-19 severity affecting both inflammatory and hemostatic pathways.

### 5.5. Molecular and Structural Considerations Involving Glutathione

Beyond its role in immune and endothelial regulation, glutathione may interact with SARS-CoV-2-related molecular processes. Structural and computational studies suggest that glutathione can interact with redox-sensitive regions of the viral spike protein, potentially influencing conformational stability or host–virus interactions [42]. While these findings remain preliminary and largely theoretical, they underscore the pervasive involvement of redox biology in SARS-CoV-2 pathophysiology.

Such molecular considerations complement broader observations that viral infections exploit host redox environments and that antioxidant depletion can facilitate viral pathogenicity. Although definitive functional implications remain to be established, these data reinforce the plausibility of glutathione as a modifier of host–virus dynamics.

### 5.6. Clinical Observations and Post-Acute Sequelae

Clinical observations extend the relevance of glutathione beyond the acute phase of COVID-19. Studies of COVID-19 survivors report persistent oxidative stress and reduced glutathione levels in subsets of patients, suggesting that redox imbalance may contribute to prolonged symptoms and delayed recovery [43]. This aligns with broader evidence that sustained oxidative stress can perpetuate inflammation, mitochondrial dysfunction, and tissue injury after acute infection.

Biomarker studies further demonstrate that glutathione-related redox measures predict disease severity, complications, and mortality [15,38,39]. These associations suggest that glutathione status reflects not only disease burden but also underlying host resilience. Importantly, these findings parallel observations in tuberculosis and other chronic infections, reinforcing the concept that glutathione depletion represents a common pathway linking diverse infectious diseases to severe outcomes in vulnerable populations.

### 5.7. Section Synthesis

In summary, glutathione emerges as a central regulator of COVID-19 pathophysiology across multiple biological domains. Glutathione deficiency predisposes individuals to severe disease by impairing redox buffering capacity, amplifying oxidative stress, disrupting immune signaling, promoting cytokine overproduction, and exacerbating endothelial and thrombotic injury [15,35,36,37,38,39,40,41]. Clinical and biomarker studies demonstrate consistent associations between reduced glutathione levels and adverse outcomes, while mechanistic analyses provide plausible pathways linking glutathione depletion to immune and vascular dysfunction [15,37,38,39,40,41,42,43].

When integrated with evidence from tuberculosis and other infectious models, these findings support a unifying framework in which glutathione availability represents a determinant of host resilience to severe infectious disease. This framework provides a strong mechanistic rationale for examining glutathione as a host-directed factor in the prevention and modulation of COVID-19, particularly in populations already burdened by metabolic, inflammatory, or oxidative stress.

## 6. Integrated Host-Directed Framework Linking Glutathione, Redox Resilience, Tuberculosis, and COVID-19 in High-Risk Populations

Tuberculosis and COVID-19 are caused by distinct pathogens and differ in transmission dynamics, tempo of illness, and clinical syndromes. Yet across both diseases, severe outcomes cluster in overlapping high-risk populations and are consistently shaped by host factors that govern immune proportionality, oxidative stress containment, endothelial integrity, and tissue repair. The preceding sections establish that glutathione depletion and broader redox imbalance can impair antimycobacterial immune control in tuberculosis through effects on macrophage function, granuloma integrity, and Th1-polarized signaling [29,30,31,32,33,34], and can worsen COVID-19 through oxidative stress amplification, cytokine-driven immune dysregulation, endothelial injury, and thromboinflammatory complications [15,35,36,37,38,39,40,41,42,43]. Section 5 synthesizes these findings into a host-directed model in which glutathione and redox signaling act as a shared vulnerability axis that helps explain why certain patients are predisposed to severe disease across different infectious threats, and why interventions that strengthen host resilience may complement pathogen-directed prevention and treatment.

### 6.1. Convergent Pathobiology Across TB and COVID-19: Immune Proportionality Under Redox Constraint

A central commonality between tuberculosis and severe COVID-19 is that pathology emerges when host responses are simultaneously excessive and ineffective. In tuberculosis, insufficient immune containment permits persistent intracellular infection, while dysregulated inflammation contributes to tissue injury and granuloma failure [29,30,31,32,33,34]. In COVID-19, severe disease reflects a similar mismatch, where antiviral responses may be poorly coordinated while inflammatory programs become amplified, leading to pulmonary injury, systemic inflammation, and vascular complications [15,35,36,37,38,39,40,41,42,43]. Redox state and glutathione availability plausibly influence both sides of this mismatch by modulating antimicrobial effector functions and constraining inflammatory escalation.

In tuberculosis models, glutathione depletion is associated with impaired containment and compromised granulomatous structure, while glutathione restoration improves immune coordination and reduces mycobacterial burden [29,30,31,32,33]. This supports the concept that adequate glutathione supports effective immunity not by indiscriminately suppressing inflammation, but by enabling immune responses that are appropriately targeted and sustained. In COVID-19, glutathione depletion is linked to redox imbalance that amplifies cytokine production, disrupts immune signaling, and promotes vascular injury, consistent with a failure of proportional immune control [15,35,36,37,38,39,40,41,42,43]. When considered together, these observations support a shared mechanistic theme: redox buffering capacity, with glutathione as a central node, may determine whether host responses remain controlled and effective versus spiraling into ineffective hyperinflammation and tissue injury [15,29,30,31,32,33,34,35,36,37,38,39,40,41,42,43].

### 6.2. The Glutathione Axis as a Shared Vulnerability Mechanism in High-Risk Hosts

High-risk populations for both tuberculosis and severe COVID-19 commonly include individuals with chronic metabolic stress, baseline inflammation, and impaired antioxidant reserves. In tuberculosis-relevant systems, glutathione deficiency promotes oxidative stress and undermines immune containment at the cellular and tissue levels, including effects on macrophage function and granuloma integrity [29,30,31,32,33,34]. In COVID-19, similar redox failure is associated with worsened inflammatory signaling and endothelial dysfunction, contributing to disease severity [15,35,36,37,38,39,40,41,42,43]. The unifying implication is that glutathione depletion is not simply a marker of severe infection but may act as a predisposing condition that lowers the threshold for immune dysregulation when the host is challenged by an acute pathogen.

When glutathione reserves are insufficient, redox-sensitive pathways may become biased toward inflammatory amplification and cellular injury, which in turn can impair pathogen clearance and promote organ damage. This framing aligns with the tuberculosis evidence showing impaired containment under glutathione depletion and improved immune coordination with supplementation [29,30,31,32,33], as well as COVID-19 evidence linking glutathione depletion to oxidative stress amplification, cytokine dysregulation, and vascular injury [15,35,36,37,38,39,40,41,42,43].

### 6.3. Inflammation, Oxidative Stress, and Tissue Remodeling: Why “Damage Biology” Matters for Both Diseases

In COVID-19, severe disease similarly involves inflammatory alveolar–capillary injury, endothelial disruption, and dysregulated repair pathways, with redox imbalance and glutathione depletion implicated in the amplification of injury mechanisms [15,35,36,37,38,39,40,41,42,43].

This convergence is important for an integrated framework because it explains why two different infections can share downstream clinical consequences in vulnerable hosts: impaired redox buffering can magnify injury responses once inflammation is triggered. In practical terms, a host-directed perspective should therefore address both pathogen containment and protection of tissues from collateral inflammatory and oxidative injury. The glutathione axis is well positioned in this respect because it is implicated in immune coordination and in limiting oxidative tissue damage across both diseases [15,29,30,31,32,33,34,35,36,37,38,39,40,41,42,43].

### 6.4. Rationale for Host-Directed Strategies: Complementing Pathogen-Directed Approaches

Pathogen-directed therapies are indispensable. Anti-tubercular regimens are curative when adhered to and drug susceptibility permits, and COVID-19 prevention and treatment has benefited from vaccines, antiviral agents, and supportive care. However, pathogen-directed approaches do not fully address the host determinants that drive severe outcomes. Host-directed strategies are designed to improve the host’s ability to tolerate infection, constrain maladaptive inflammation, preserve organ function, and enhance effective immune responses. Contemporary host-directed therapy frameworks emphasize that targeting host factors can be particularly valuable when disease severity is dominated by immune dysregulation and tissue injury, or when high-risk hosts have baseline vulnerabilities that persist across different infections [44].

In a synthesis section, this is the key conceptual bridge: the same host pathways that predispose a patient to severe COVID-19 may also predispose them to poor outcomes in tuberculosis, and host-directed approaches can be structured to improve resilience across infectious threats rather than only treating a single pathogen in isolation [44,45,46,47].

The host-directed immunotherapy literature further supports this approach as broadly applicable across pathogens. Conceptual and translational discussions of host-directed immunotherapy to fight infectious diseases emphasize the potential to augment protective immunity while mitigating damaging immune responses, thereby improving outcomes in both viral and bacterial infections [47].

Mechanistically, glutathione’s relevance extends beyond antioxidant scavenging. Glutathione regulates redox-sensitive signaling pathways that shape innate immune activation, cytokine production, and inflammatory resolution. Redox signaling is increasingly understood as a core regulator of innate immunity and inflammation, influencing the intensity, duration, and tissue consequences of immune activation [48].

Both TB and severe COVID-19 involve not only pathogen control but also host tissue damage and repair. Tuberculosis pathology is tightly linked to inflammatory remodeling in the lungs, including fibrosis and cavitation in advanced disease, processes that are influenced by persistent inflammation and oxidative stress. Work examining the interplay between systemic inflammation, oxidative stress, and tissue remodeling in tuberculosis reinforces the concept that redox imbalance participates in downstream structural lung injury, not merely upstream immune dysregulation [49].

### 6.5. Positioning Glutathione Within Host-Directed Immunotherapy and Redox-Guided Interventions

Within host-directed frameworks, glutathione and redox signaling occupy a practical niche because they sit upstream of multiple downstream disease drivers: cytokine amplification, macrophage antimicrobial competence, endothelial integrity, and tissue remodeling [15,29,30,31,32,33,34,35,36,37,38,39,40,41,42,43,48,49]. Host-directed immunotherapy frameworks for viral infections emphasize that host pathways shaping immune activation, inflammatory magnitude, and tissue injury can be targeted to improve outcomes, particularly in severe disease states characterized by dysregulated inflammation [45]. When integrated with glutathione-centered evidence from COVID-19 and TB, this suggests a redox-guided host-directed approach could plausibly include strategies aimed at restoring glutathione availability, improving redox buffering, and constraining oxidative amplification of inflammation, while supporting effective antimicrobial immunity [15,29,30,31,32,33,34,35,36,37,38,39,40,41,42,43,48].

Importantly, the evidence base summarized in Section 3 and Section 4 does not justify simplistic claims that glutathione “prevents” either disease. Rather, it supports a more defensible and clinically credible statement: glutathione status may influence susceptibility to severe disease and modulate downstream immune and vascular pathology in high-risk populations. In tuberculosis, glutathione supplementation studies demonstrate improved immunologic patterns and reduced mycobacterial burden in experimental systems [30,31,32,33], while depletion studies demonstrate worsened containment [29]. In COVID-19, clinical and mechanistic analyses link glutathione depletion to oxidative stress, immune dysregulation, and endothelial injury associated with severe outcomes [15,35,36,37,38,39,40,41,42,43]. A synthesis section should therefore treat glutathione as a host resilience factor with mechanistic plausibility and supportive experimental and clinical correlates, and place it within the broader host-directed immunotherapy landscape rather than presenting it as a standalone cure.

### 6.6. Implications for High-Risk Populations and Prevention-Oriented Strategy Design

The integrated framework has practical implications for how high-risk populations are conceptualized and prioritized. Patients with metabolic disease, chronic inflammatory burden, or reduced antioxidant reserves can be viewed as having diminished redox resilience, which may increase vulnerability to severe outcomes across pathogens. This supports prevention-oriented strategies that combine traditional infectious disease prevention measures with targeted efforts to improve host resilience, particularly in populations where baseline vulnerabilities are modifiable. In the tuberculosis context, this could mean identifying high-risk hosts where glutathione depletion and immune dysregulation intersect to impair granuloma containment [29,30,31,32,33,34]. In COVID-19, it could mean identifying hosts where glutathione depletion aligns with redox imbalance, cytokine amplification, and endothelial dysfunction [15,35,36,37,38,39,40,41,42,43].

Host-directed therapy frameworks emphasize that the future of infectious disease management will likely require layered strategies that integrate pathogen-directed tools with host-centered interventions tailored to risk profiles, rather than a one-size-fits-all approach [44]. The host-directed immunotherapy literature similarly supports cross-pathogen approaches that strengthen protective immune responses and reduce collateral injury [45,46,47]. Redox signaling work reinforces that the immune system’s set points are sensitive to redox balance, making this axis a plausible target for improving immune proportionality and reducing damaging inflammation [48]. The tuberculosis remodeling literature adds that downstream tissue injury pathways, influenced by inflammation and oxidative stress, are integral to long-term outcomes and therefore should be considered part of the host-directed target space [49]. Together, these sources support a synthesis that is mechanistically coherent and strategically aligned with prevention and risk reduction in high-risk populations.

### 6.7. Section Synthesis

In summary, tuberculosis and COVID-19 converge on host determinants that shape immune proportionality, oxidative stress containment, endothelial integrity, and tissue repair. Across both diseases, glutathione depletion and redox imbalance are consistently positioned as contributors to immune dysfunction and tissue injury in vulnerable hosts, supported by experimental and translational evidence in tuberculosis [29,30,31,32,33,34] and mechanistic and clinical evidence in COVID-19 [15,35,36,37,38,39,40,41,42,43]. Host-directed therapy and immunotherapy frameworks provide a conceptual scaffold for integrating these findings into a prevention- and resilience-oriented strategy that complements pathogen-directed interventions [44,45,46,47]. Redox signaling scholarship further supports redox balance as a regulator of innate immunity and inflammation, providing mechanistic plausibility for redox-guided host-directed approaches [48]. Finally, tuberculosis-specific evidence linking inflammation and oxidative stress to tissue remodeling underscores that redox biology influences not only early immune responses but also downstream structural outcomes that determine long-term morbidity [49].

This integrated framework sets up a logical transition to final sections addressing practical implications, limitations, and testable hypotheses. In particular, it motivates carefully designed studies in high-risk populations to determine whether glutathione status can serve as a risk stratification marker and whether redox-guided host-directed strategies can meaningfully reduce severe outcomes across distinct infectious threats without compromising pathogen control [15,29,30,31,32,33,34,35,36,37,38,39,40,41,42,43,44,45,46,47,48,49].

## 7. Integration of Glutathione-Based Redox Biomarkers in Preclinical Evaluation of Novel Anti-Tuberculosis Therapeutics

In recent tuberculosis research, glutathione and its precursor N-acetylcysteine have increasingly been evaluated as biologically meaningful indicators of host redox status and immune competence during antimicrobial therapy. In the RIPENACTB randomized controlled trial, hospitalized patients with HIV-associated pulmonary tuberculosis were treated with adjunctive oral N-acetylcysteine in addition to standard anti-tubercular therapy, and detailed biochemical analyses demonstrated significant reductions in systemic oxidative stress markers, including malondialdehyde and protein carbonyl content, alongside increases in intracellular reduced glutathione levels within peripheral blood mononuclear cells. These biochemical changes were accompanied by normalization of inflammatory mediators, improved macrophage antimicrobial function, and enhanced T lymphocyte responsiveness, suggesting that restoration of glutathione-dependent redox balance was closely linked to improved host control of Mycobacterium tuberculosis during active infection and treatment [50].

A recent prospective clinical study evaluating adjunctive N-acetylcysteine in adults with pulmonary tuberculosis further confirmed the clinical relevance of glutathione modulation. In this investigation, patients receiving NAC alongside first-line therapy demonstrated progressive increases in plasma and intracellular glutathione concentrations over the course of treatment, as measured by enzymatic recycling assays, compared with controls receiving anti-tubercular therapy alone. These biochemical improvements were associated with accelerated recovery of forced expiratory volume and forced vital capacity, reduced radiographic progression, and earlier sputum culture conversion. Importantly, longitudinal analysis showed that patients with the greatest increases in glutathione levels exhibited the most consistent improvements in lung function and inflammatory marker normalization, supporting the use of glutathione as a surrogate indicator of therapeutic response in vivo [51].

Comprehensive mechanistic and translational evidence supporting this relationship is summarized in a detailed review of N-acetylcysteine as an adjunctive tuberculosis therapy. This analysis collated data from animal models, in vitro macrophage infection systems, and early-phase clinical trials, demonstrating that NAC supplementation increases intracellular cysteine availability and drives glutathione synthesis in immune cells. Enhanced glutathione availability was shown to improve phagolysosomal maturation, promote nitric oxide-mediated antimycobacterial activity, and regulate nuclear factor kappa B signaling pathways involved in cytokine production. The review further described how restoration of redox homeostasis reduces excessive tissue damage while preserving bactericidal immune responses, emphasizing that glutathione status can be quantitatively monitored as a pharmacodynamic marker of host-directed therapeutic efficacy [52].

The broader pathophysiological relevance of oxidative stress and glutathione depletion in tuberculosis is extensively documented in a systematic analysis of redox biology in human TB. This work demonstrated that active tuberculosis is consistently associated with reduced plasma and cellular glutathione levels, increased lipid peroxidation products, impaired antioxidant enzyme activity, and dysregulated mitochondrial function in immune cells. Using both clinical samples and experimental models, the authors showed that oxidative stress promotes macrophage dysfunction, enhances mycobacterial survival, and contributes to progressive lung injury. Importantly, therapeutic interventions that restored glutathione pools were associated with improved immune signaling, reduced granulomatous necrosis, and enhanced responsiveness to antimicrobial drugs, providing a mechanistic rationale for using glutathione measurements as indicators of therapeutic effectiveness [53].

Direct evidence that glutathione-related oxidative markers can be applied to evaluate newly synthesized anti-tuberculosis drug candidates is provided by a preclinical toxicology and efficacy study of novel nitrofuranyl amide compounds. In this investigation, experimental agents were administered to rodent models, and comprehensive biochemical profiling was performed to assess systemic and tissue-specific oxidative stress. Treated animals demonstrated dose-dependent changes in hepatic and pulmonary reduced and oxidized glutathione ratios, glutathione peroxidase activity, and superoxide dismutase expression. These redox parameters were analyzed alongside histopathologic findings, bacterial load measurements, and pharmacokinetic data. Compounds associated with preservation of glutathione homeostasis and reduced lipid peroxidation exhibited superior safety profiles and enhanced antimycobacterial activity, whereas agents inducing glutathione depletion were linked to increased tissue toxicity. This study illustrates how glutathione-based biomarkers can be incorporated into preclinical screening pipelines to evaluate both the therapeutic efficacy and host toxicity of novel tuberculosis drug candidates [54].

## 8. Conclusions

This review examined the role of glutathione and redox regulation as unifying host determinants of immune dysfunction and disease severity in tuberculosis and COVID-19. Across experimental, translational, and clinical studies, consistent evidence indicates that impaired glutathione availability is associated with dysregulated immune signaling, excessive oxidative stress, and increased tissue injury, whereas restoration of redox balance supports antimicrobial defense, inflammatory proportionality, and preservation of organ function.

Mechanistic data demonstrate that glutathione functions as an active regulator of immune metabolism, signaling fidelity, and effector programming. Adequate glutathione synthesis is required for sustained T cell activation and differentiation, while in innate immunity it shapes macrophage polarization, cytokine production, and phagolysosomal competence. At the molecular level, reversible S-glutathionylation provides a direct mechanism linking redox status to immune and metabolic pathways. These interconnected processes establish glutathione as a determinant of immune coordination rather than a secondary marker of oxidative stress.

Disease-specific evidence reinforces this framework. In tuberculosis, glutathione deficiency compromises granuloma integrity, weakens Th1-mediated containment, and promotes persistent infection. In COVID-19, depletion amplifies oxidative stress, disrupts antiviral signaling, and exacerbates endothelial and inflammatory injury. Despite distinct pathogen biology, both diseases converge on shared vulnerabilities governed by redox imbalance.

Clinical and translational studies demonstrate that glutathione status is modifiable and responsive to interventions such as N-acetylcysteine and liposomal glutathione. Preclinical studies further support the use of glutathione-related biomarkers in evaluating therapeutic efficacy and toxicity. Together, these findings support integration of redox-guided strategies into host-directed therapeutic frameworks.

Future studies should prioritize standardized biomarker assessment and well-controlled clinical trials to determine whether glutathione-guided interventions can improve outcomes in high-risk populations. Overall, this synthesis supports glutathione as a biologically grounded, clinically relevant host factor with potential to enhance resilience against severe infectious disease.

## Figures and Tables

**Table 1:** Summary table of macrophage redox control in TB.

Aspect	Evidence Source	Key Mechanism	Immune Effect	Relevance to TB Pathophysiology
Macrophage redox balance	GSH depletion models [29]	Reduced intracellular glutathione increases oxidative stress	Impaired phagolysosomal function and intracellular signaling	Weakens early antimicrobial defense mechanisms
Oxidative stress amplification	Murine and cellular studies [29]	Excess accumulation of reactive oxygen species	Disruption of antimicrobial effector pathways	Promotes intracellular *Mycobacterium tuberculosis* survival
Granuloma formation	Murine tuberculosis models [29]	Altered immune cell organization and recruitment	Defective granuloma architecture	Reduces physical and immunologic containment
Tissue-level immunity	In vivo infection studies [29]	Redox imbalance affects cellular coordination	Increased bacterial persistence	Facilitates progressive disease development
High-risk host vulnerability	Translational and population analyses	Elevated baseline oxidative burden	Heightened macrophage dysfunction	Explains increased severity in metabolically stressed populations

## Data Availability

No new data were created or analyzed in this study. Data sharing is not applicable to this article.
